# Comparison of pharmacokinetics of omega-3 fatty acid supplements in monoacylglycerol or ethyl ester in humans: a randomized controlled trial

**DOI:** 10.1038/s41430-020-00767-4

**Published:** 2020-10-03

**Authors:** Laurie Chevalier, Mélanie Plourde

**Affiliations:** 1 Centre de Recherche sur le Vieillissement, Centre Intégré Universitaire de Santé et Services Sociaux de l’Estrie-Centre Hospitalier Universitaire de Sherbrooke, Sherbrooke, QC Canada; 2 Faculté de Médecine et des Sciences de la Santé, Université de Sherbrooke, Sherbrooke, QC Canada

**Keywords:** Fat metabolism, Fatty acids

## Abstract

**Background:**

A diet low in omega-3 fatty acids (n-3 FA) results in low plasma concentrations of docosahexaenoic acid (DHA) and eicosapentaenoic acid (EPA), the two main long chain n-3 FA. n-3 FA supplements on the market are esterified in triglycerides (TG) or ethyl ester (EE); the latter is absorbed less than other esterification forms. The objective of this study was to test and compare the pharmacokinetics of n-3 FA esterified in monoacylglycerides (MAG), a predigested form, with the EE form.

**Methods:**

This study was a randomized, double-blind, crossover, controlled, clinical trial. Ten men and ten women between 18 and 60 years old were recruited. Participants received a single oral dose of 3 g of n-3 FA esterified in EE or MAG. Eleven blood samples were collected over 24 h post-dose. Plasma total lipids were extracted, methylated, and analyzed using gas chromatography.

**Results:**

After receiving the MAG form, plasma EPA and DHA peaked at a concentration 3 and 2.5 times higher, respectively, than with the EE form. When provided in MAG form, n-3 FA plasma concentration during the absorption phase was on average 3–5 times higher than in EE form. When n-3 FAs were provided esterified in MAG, their concentration 24 h post-dose was higher than in EE. Males had a lower n-3 FA plasma concentration than females when n-3 FAs were provided in EE but there was no sexe difference when provided in MAG.

**Conclusions:**

Plasma concentration of DHA and EPA was higher when provided in MAG than EE form.

## Background

Dietary fat intake and the type of fat consumed changed dramatically in the last century [1]. For example, today’s diet contains more saturated fat, *trans* fat and omega-6 polyunsaturated fat and less omega-3 fatty acids (n-3 FA), especially eicosapentaenoic acid (EPA) and docosahexaenoic acid (DHA) [2]. A meta-analysis of 298 studies found that North Americans had <4% (wt%) EPA and DHA in erythrocytes or erythrocyte equivalents [3] whereas expert groups recommend at least 8% EPA and DHA blood levels [4]. Humans cannot synthesize n-3 FAs since they lack the enzyme for generating an n-3 double bond in fatty acids. Therefore, dietary intake of n-3 FA is required to increase blood levels.

n-3 FA intake has shown various health benefits. In the body, n-3 FAs inserted into cell membranes increase membrane fluidity. Their hydrolysis by phospholipase from the membrane can generate an anti-inflammatory response via, among other things, the formation of resolvins and the reduction of pro-inflammatory metabolites [5, 6]. Also, DHA along with arachidonic acid are the most abundant polyunsaturated fatty acids in brain membranes. DHA accounts for between 11 and 34% of the fatty acids in the cerebral cortex [7] and is mainly concentrated in the neurons [8]. A recent meta-analysis in humans showed that, among 299 blood metabolites, higher plasma DHA levels were associated with a lower risk of all-cause dementia and Alzheimer’s disease [9]. Furthermore, in some studies EPA and DHA supplementation has been shown to benefit cardiovascular health [10–12]. Having more than 8% of EPA and DHA in erythrocyte total lipids compared to 4% was associated with 90% fewer cardiovascular deaths [13].

In addition to a low intake of n-3 FA, the Western diet is rich in n-6 fatty acids [2]. This creates an imbalance in the n-6/n-3 ratio, which is associated with various diseases, such as cardiovascular and inflammatory, and some cancers [2, 6]. A simple and quick way to rebalance the n-6/n-3 ratio is to consume more n-3 FA in the form of supplements [14]. The most commonly studied forms of n-3 FA are naturally occurring and reconstituted triglycerides (TG), ethyl ester (EE), and free fatty acids (FFA). Single dose studies evaluating the pharmacokinetics of n-3 FA esterified in different forms reported that the most bioavailable form of n-3 FA is when provided in FFA > TG > EE [15]. However, FFA are highly susceptible to peroxidation [16] and therefore less stable over time, which explains why n-3 FA are usually esterified in EE or TG. With the EE and TG forms, however, there are side effects since they require pancreatic lipases in the digestion process whereas FFA and monoacylglycerol (MAG) forms are considered as being in a predigested form. The most common reported side effects of EE and TG n-3 FA are gastrointestinal discomfort, nausea, and gastric reflux [17]. One potential solution is to provide n-3 FA in the MAG predigested form. A previous study reported that MAG n-3 FA can be efficiently absorbed in people with fat digestion issues [18]. The objective of this study was to evaluate whether plasma concentration of MAG n-3 FA is higher than an EE form of n-3 FA in a healthy population. We hypothesized that over 24 h, higher n-3 FA plasma concentrations would be reached when EPA and DHA were provided esterified in MAG rather than in EE.

## Methods

### Inclusion and exclusion criteria

We recruited ten males and ten females between 18 and 60 years old at Diex Research, a private clinical research company located in Sherbrooke, Quebec, Canada. Participants provided a medical history and underwent a physical examination and blood test before inclusion. Body mass index had to be between 19 and 30 kg/m^2^. Individuals had to refrain from participating in other clinical studies involving experimental drugs for at least 30 days. People were excluded from this study if they had consumed natural health products containing n-3 FAs in the previous 6 months, if they had allergies or intolerances to fish, or if they had a special diet. People were also excluded if they were smokers, or if they reported regularly consuming drugs or alcohol in the previous 12 months (females > 10 drinks/week, males > 14 drinks/week). Anyone who had donated more than 500 mL of blood in the previous 56 days was excluded. People presenting cardiovascular, pulmonary, hematological, neurological, psychiatric, endocrine, or immunological problems as well as gastrointestinal tract, liver or kidney disease, or other conditions that could affect the absorption of lipids were excluded from this study. Also excluded were people with hypothyroidism, moderate-to-severe lipidemia (total cholesterol ≤ 240 mg/dl; LDL ≤ 160 mg/dl; TG ≤ 199 mg/dl), systolic blood pressure above 160 mmHg and diastolic blood pressure above 95 mmHg, or cardiac output at rest <40 beats/min or >100 beats/min.

Females with childbearing potential were tested for serum hCG at screening and urinary hCG before the supplementation. Positive hCG led to participant exclusion or withdrawal. Females of childbearing potential had to have adequate contraception, i.e., they had to use a condom as well as one of the following methods: spermicide, diaphragm with spermicide, hormone-free IUD, or other. Females using hormonal contraception were excluded from this study. Females between 45 and 60 years old had to have had amenorrhea for more than 2 years and a negative hCG or must have had a bilateral tubal ligation, hysterectomy, or oophectomy to be included in this study. All participants provided written informed consent. This trial is registered at clinicaltrials.gov under number NCT04382027.

### Study design

**Fig. 1:**
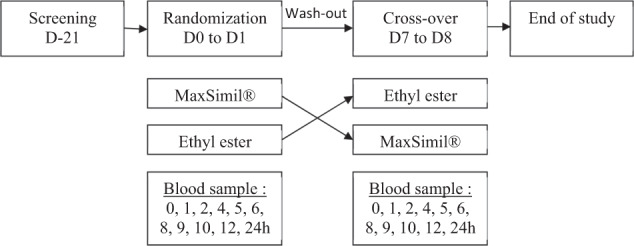
Study design of the randomized clinial trial. Screening was performed at day minus 21 days of the end of the trial. Randomization was performed at day 0 and lasted until day 1 of the trial and participants tested the second supplement at day 7 untils day 8 (cross-over). The two supplements were MaxSimil (R), a monoglycerides-based omega-3 supplement, and omega-3 fatty acids esterified in ethyl esters. For each suplement tested, a blood sample was collected post single dose intake at the times detailed in the blood sample box.

This study employed a randomized, double-blind, crossover design. The two treatments were monoglyceride rich oil (MAG) (MaxSimil^®^ 3020) and an ethyl ester form (EE). The fatty acid profile of the capsules is provided in Table 1. Randomization was performed by a biostatistics expert. Each participant was assigned a random number in chronological order of enrollment. This number determined the sequence order of treatment, i.e., MAG then EE, or EE then MAG. No participant was excluded from the study following randomization. The supplements were prepared by a nonblinded member of the research team based on the randomization table. With the exception of this person, participants, nurses and members of the research team were all blinded. Participants received a single oral dose of 6000 mg of lipids (6 × 1000 mg capsules) containing a total of 3000 mg n-3 FA (1800 mg EPA and 1200 mg DHA). Both supplements were softgels and they were identical in color, shape, and size. Participants had 60 s to ingest 250 mL of water and the six capsules. The first blood sample was taken after the participants had fasted for 12 h and before intake of the supplements. Thereafter, ten blood samples of 5 mL each were collected 1, 2, 4, 5, 6, 8, 9, 10, 12, and 24 h after the single dose intake. The study design is shown in Fig. 1. The blood samples were centrifuged at 1700 g for 10 min at 4 °C. The plasma was separated from white and red blood cells and stored at −80 °C until further analysis.

**Table 1:** Fatty acid profile of the monoacylglycerol (MAG) and ethyl ester (EE) capsules.

Fatty acids	MAG (%)	EE (%)	Fatty acids	MAG (%)	EE (%)
C14:0	0.6	0.6	C20:1	1.7	1.7
C15:0	0.1	0.1	C20:2	0.3	0.4
C16:0	3.3	3.4	C21:0	0.1	0.1
C16:1	1.3	1.3	C20:3 n-6	0.3	0.3
C17:0	0.2	0.2	C20:4 n-6	1.3	1.3
C17:1	0.2	0.2	C20:3 n-3	0.2	0.2
C18:0	3.3	3.2	C22:0	0.2	0.2
C18:1 n-9 (t)	0.0	0.1	**C20:5 n-3**	**33.5**	**33.4**
C18:1 n-9 (c)	6.8	6.8	C22:2	0.0	0.1
C18:2 n-6 (c)	0.9	1.0	C23:0	0.0	0.0
C19:0	0.1	0.1	C24:0	0.3	0.3
C18:3 n-6	0.2	0.2	C22:5 n-3	4.4	4.4
C18:3 n-3	0.8	0.8	C24:1	0.5	0.5
C20:0	0.4	0.4	**C22:6 n-3**	**24.6**	**24.6**

Bold fatty acids indicate the two main long chain omega-3 fatty acids of the supplements.

The participants’ diet was standardized during the 24-h study period. Although meal-taking influences the pharmacokinetics of supplements, we made the decision to test the supplements with meals instead of fasting because we wanted to assess the n-3 FA pharmacokinetic profile in a situation as close as possible to reality. The meals were served 5 min after the blood sample collections at *t* = 0 h, 4 h and 9 h, and the participants had 25 min to eat. The supplement was consumed with a standardized breakfast of 366 kcal (60% carbohydrate, 20% fat, and 20% protein). The total daily calorie intake was 2150 ± 196 kcal for males and 2181 ± 196 kcal for females (59% carbohydrate, 25% fat, and 16.0% protein).

### Fatty acids extraction and analysis

Plasma samples were thawed at room temperature and sonicated at 30% amplitude for 15 s. A precise amount of 0.042 mg of triheptadecanoin (C17: 0 in TG form, Nu-Chek Prep, USA) was added to 100 μl of plasma to allow the quantification of fatty acids. Lipids were extracted from the plasma according to Folch et al.’s method [19]. Next, the fatty acids were saponified with KOH–methanol (5.6%) for 1 h at 90 °C and the ionized fatty acids were isolated by liquid–liquid extraction with 5 mL of hexane and 2 mL of saline (0.9%). Then the fatty acids were collected in the aqueous phase and protonated with 300 μl of HCl. A second liquid–liquid extraction was then performed with hexane and saline and the fatty acid was collected in the organic phase. Hexane was evaporated before the fatty acids were methylated with 3 mL of 14% BF_3_–methanol (Aldrich Chemistry) at 90 °C for 30 min. The methylated fatty acids were recovered in hexane and concentrated in 1.5 mL of hexane before analysis.

The methylated fatty acids were analyzed using an Agilent 6890 series gas chromatograph equipped with a hydrogen flame ionization detector. 1 μl of the sample was injected in splitless mode into the SGE BPX-70 silica column (70% cyanopropyl polysilphenylsiloxane) at a temperature of 50 °C. The temperature was then increased to 170 °C with a gradient of 20 °C/min and held at this temperature for 15 min. Then the temperature was increased to 210 °C with a gradient of 5 °C/min. Retention times of the samples were compared to the identified peaks obtained from Nu-Chek Prep standards. OpenLab CDS ChemStation was used to perform the chromatogram analyses.

### Statistical analysis

To determine the size of our sample, we conducted a literature review on the pharmacokinetics of n-3 FA supplements. Since the MAG supplement has not been widely studied, there was no similar study to use for our sample size calculation. Therefore, we examined studies of the pharmacokinetics of n-3 FA esterified in different forms. Some had a sample size between 8 and 20 people [20–22] while others had bigger samples [23, 24]. We decided to base our sample size on the smallest sample as was done previously [25, 26]. Hence, we recruited 20 participants (10 men and 10 women) for this study. Once the study was completed, we calculated the effect size using the area under the curve (AUC) 0–5 h and 0–24 h for EPA, DHA, and EPA + DHA. Since our standard deviations were similar and our sample size was equal for both groups, we chose Cohen’s *d* test and reported values in a range from 2 to 5, which is considered a large effect size. This test confirmed that with our sample size of 20 subjects, we had enough statistical power to detect significant differences between our two n-3 FA formulations.

The primary outcome metrics used in this study were the plasma concentration of EPA and DHA during the absorption phase, which is the AUC between 0 and 5 h, and the plasma concentration of EPA and DHA over 24 h, which is the AUC between 0 and 24 h. The secondary outcome included the maximum concentration (Cmax) and the concentration of EPA and DHA in the plasma at 24 h. All statistical analyses were performed with either GraphPad Prism 7.03 software or IBM SPSS Statistics version 25. The pharmacokinetic curves for each group were obtained from the means and standard deviations for each time point. Each value represents the delta over baseline (*Y* = *Y*_x_ − *Y*_0_) since we wanted to evaluate the increase in EPA + DHA over the initial plasma level after taking the supplement.

Each variable was tested for normality using the Shapiro–Wilk test and for homogeneity of variances using Levene’s test. A paired *t* test was performed when variables met both conditions. The tested variables were: 5-h AUC of the % area of EPA and EPA + DHA; 5-h AUC of EPA, DHA, and EPA + DHA concentration; 24-h AUC of the % area of EPA, DHA, and EPA + DHA; 24-h AUC of DHA concentration; maximum % area of EPA and EPA + DHA; Cmax of EPA, DHA, EPA + DHA; % area at 24 h of EPA, DHA, and EPA + DHA; and concentration of DHA at 24 h. For variables not meeting at least one of these conditions (normality and homogeneity of variances), we performed a paired sample Wilcoxon test. (These variables were: 24-h AUC of EPA and EPA + DHA concentration; 5-h AUC of the % area of DHA; concentration of EPA and EPA + DHA at 24 h; and maximum % area of DHA.) Statistical significance was set at *α* = 0.05.

## Results

The study included ten males and ten females with an average age of 33 ± 11 years and 43 ± 11 years, respectively (*p* = 0.0636). Average BMI was 23.6 ± 2.6 kg/m^2^ for males and 24.0 ± 11 kg/m^2^ for females. The participants recruited were 90% Caucasian; 10% of males were Hispanic American and 10% of females were Latin American (Table 2). There were no apparent differences between males and females on the characteristics tested.

**Table 2:** Anthropometric characteristics of the participants.

	Males	Females	*P* value
Sample size	10	10	–
Age, years	33 ± 11	43 ± 11	0.0636
BMI, kg/m^2^	23.6 ± 2.6	24.0 ± 2.7	0.7771
Ethnic group	9 Caucasians 1 Hispanic American	9 Caucasians 1 Latin American	<0.05

Data are means ± standard deviation. *BMI* body mass index, ethnic group was self declared.

### MAG vs EE omega-3 supplements

**Fig. 2:**
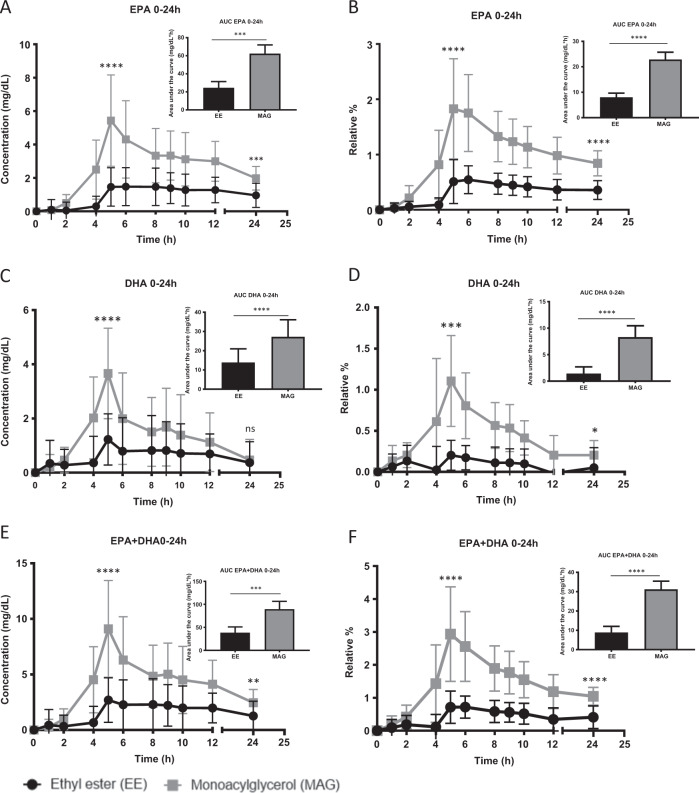
Pharmacokinetics of eicosapentaenoic acid and docosahexaenoic acid with monoacylglyceride (gray) or ethyl ester (black) supplement. **a** Concentration (mg/dL) of plasma eicosapentaenoic acid (EPA) in total lipids over 24 h; **b** Relative % of plasma EPA in total lipids over 24 h; **c** Concentration (mg/dL) of plasma docosahexaenoic acid (DHA) in total lipids over 24 h; **d** Relative % of plasma DHA in total lipids over 24 h; **e** Concentration (mg/dL) of plasma EPA + DHA in total lipids over 24 h; **f** Relative % of plasma EPA + DHA in total lipids over 24 h. All results are expressed as mean ± SD (*n* = 20). AUC = area under the curve. ns *p* value > 0.05 (*t*-test), *p value = 0.0136 (*t*-test), **p value = 0.005 (Wilcoxon test), ***p value < 0.001 (Wilcoxon test), ****p value < 0.0001 (*t*-test).

Pharmacokinetics of the two supplements are presented in Fig. 2. The absorption phase of the supplement was defined as 0–5 h after supplement intake. When provided in MAG form, plasma concentrations of EPA, DHA, and EPA + DHA during the absorption phase were respectively five, three, and four times higher than in EE form (*p* < 0.0001) (Fig. 2a-c-e). The % of EPA, DHA, and EPA + DHA relative to other plasma fatty acids were on average five times higher when provided as MAG vs. EE (*p* < 0.0001, *p* = 0.001, *p* < 0.0001) (Fig. 2b–d). With respect to 24-h n-3 FA plasma concentrations, they were on average two times higher when provided as MAG compared to EE (Fig. 2a-c-e). When expressing the results in relative % to other FAs, 24-h plasma concentrations of EPA, DHA, and EPA + DHA were respectively 2.8, 5.8, and 3.5 times higher with the MAG supplement than the EE form (*p* < 0.0001) (Fig. 2b-c-d). Also, the maximum plasma concentrations (mg/dL) and relative % to other fatty acids of EPA, DHA, and EPA + DHA were on average 2.7 times and 4 times higher, respectively, with the MAG formulation than the EE form (*p* < 0.0001) (Fig. 2). Twenty-four hours after providing the single dose of n-3 in either MAG or EE, the residual concentration of EPA was two times higher when provided as MAG than EE (*p* < 0.0001) (Fig. 2a). When expressed in relative % to other fatty acids, residual n-3 FA in the blood after the single dose n-3 were two to four times higher when provided as MAG rather than EE (Fig. 2b-d-f). Data are provided in Table 3 and Supplementary Information File 1.

**Table 3:** Pharmacokinetic parameters of plasma eicosapentaenoic acid + docosahexaenoic acid after supplementation with monoacylglycerol and ethyl ester.

Pharmacokinetic parameters	Concentration EPA + DHA		% area EPA + DHA	
	Mean ± SD	*P* value	Mean ± SD	*P* value
Cmax				
—EE	3.6 ± 1.9	<0.0001	0.8 ± 0.4	<0.0001
—MAG	9.7 ± 4.0		3.2 ± 1.2	
Concentration *T* = 24 h				
—EE	1.3 ± 1.3	0.005	0.4 ± 0.4	<0.0001
—MAG	2.5 ± 1.2		1.0 ± 0.3	
AUC 0–5 h				
—EE	3.3 ± 2.5	<0.0001	0.8 ± 0.6	<0.0001
—MAG	13.0 ± 4.1		4.4 ± 1.6	
AUC 0–24 h				
—EE	38.4 ± 12.4	<0.0001	8.9 ± 3.2	<0.0001
—MAG	89.6 ± 17.0		31.2 ± 4.2	

*EPA* eicosapentaenoic acid, *DHA* docosahexaenoic acid, *EE* ethyl ester, *MAG* monoacylglycerol,

*SD* standard deviation, *Cmax* maximum concentration (mg/dL), *Concentration T* = *24* *h* concentration of FAs at 24 h post supplementation (mg/dL), *AUC 0–5* *h* area under the curve from time 0 to 5 h post supplementation (mg/dL*h), *AUC 0–24* *h* area under the curve from time 0 to 4 h post supplementation (mg/dL*h).

### Comparisons of the pharmacokinetics in males and females

**Fig. 3:**
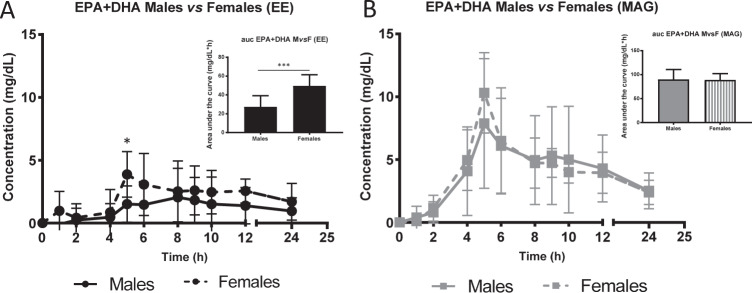
Pharmacokinetics of eicosapentaenoic acid plus docosahexaenoic acid between sexes after ethyl ester or monoacylglyceride supplementation. Females are represented by dotted lines and males by the continuous line. **a** Concentration (mg/dL) of plasma eicosapentaenoic acid + docosahexaenoic acid (EPA + DHA) in total lipids over 24 h in ethyl ester form; **b** Concentration (mg/dL) of plasma EPA + DHA in total lipids over 24 h in monoacylglyceride form. All results are expressed as mean ± SD (*n* = 20). AUC = area under the curve. ns *p* value > 0.05 (*t*-test), *p value = 0.0295 (*t*-test), ***p value = 0.0006 (*t*-test).

We compared the plasma concentrations of EPA + DHA between males and females after consuming the single oral dose of the MAG or EE supplement (Fig. 3). The results show that following supplementation with the EE form, females had a plasma concentration 2.7 times higher during the absorption phase (*p* = 0.0111), 1.8 times higher 24-h plasma concentration (*p* = 0.0006) and 1.7 times higher maximum concentration (*p* = 0.0295) compared to males (Fig. 3a). However, apart from having significantly higher plasma EPA and DHA concentrations with MAG than EE, there was no significant difference between males and females (Fig. 3b). The pharmacokinetic curves for EPA or DHA alone were similar to the EPA + DHA curve. Therefore, only the pharmacokinetic curves for EPA + DHA are shown in Fig. 3. Data are also provided in Supplementary Information File 2.

## Discussion

This study compares the pharmacokinetics of two different forms of n-3 FA supplements, i.e., MAG form and EE form. Our results indicate that a higher plasma concentration of n-3 FA was reached in the absorption phase and over 24 h when provided as MAG instead of EE, which confirms our hypothesis. To explain this result, it is important to remember that the level of fatty acids in the plasma is a balance between the uptake and release of fatty acids from organs and tissues. The first step in this process is absorption. When n-3 FA were provided esterified in MAG, the hydrolysis of FA by pancreatic lipases was not required [27, 28], which potentially led to a higher uptake of EPA and DHA by the enterocytes compared to the EE form as seen from the pharmacokinetic curves. EE requires enzymatic breakdown to generate non-esterified FA [23] but the efficiency of this process is poor because of its low affinity with pancreatic lipase [21]. After being generated, non-esterified FA can cross the cell membrane by passive diffusion or by using a fatty acid transporter such as FATP4 or CD36 [27, 28]. Our results support this physiological pathway since the slope of the increase within 5 h post-dose is sharper with the MAG supplement than the EE supplement. Another explanation is greater clearance of EE-EPA + DHA from the bloodstream. EPA and DHA can be converted to other signaling molecules but we did not analyze these molecules in the plasma. Previous studies by our group using uniformly labeled carbon 13 DHA and EPA esterified in methyl esters showed similar kinetic curves to the ones we obtained here [29–31], indicating that once methyl and ethyl esters enter the bloodstream, they are not rapidly cleared. Another potential explanation is that with the MAG form, EPA + DHA remain in the bloodstream for a longer period, which delays their entry into organs and tissues. Whether this is good or not is unclear but studies evaluating the link between plasma EPA + DHA levels and risk of chronic diseases usually point to less risk of chronic diseases in people with higher EPA + DHA plasma levels [9, 11, 32, 33].

Our results are difficult to compare directly to other studies since most pharmacokinetic studies with n-3 FA supplements involved FFA, TG, EE, and sometimes phospholipid (PL) forms. The majority of short-term studies demonstrated that n-3 FA supplementation esterified in EE had lower plasma concentrations and relative % of n-3 FAs compared to PL, TG, and FFA forms [15, 21, 22, 34]. One study reported the pharmacodynamics of n-3 FA esterified in MAG provided to overweight or obese participants for 21 days. They reported that plasma concentration of EPA was 56% higher (*p* < 0.0001) when provided as MAG compared to TG but only in those taking a pancreatic lipase inhibitor [18]. These results indicate that pancreatic lipases are important for fat absorption, and MAG could be a solution to circumvent lower levels or poorer efficiency of these lipases. In general, previous pharmacokinetic studies in humans showed that n-3 FA plasma concentrations increased less when n-3 FA were provided esterified in EE compared to the TG esterified form, and we provide evidence here that plasma n-3 FA concentrations increase when they are provided esterified in MAG.

Other studies of pharmacokinetics have been conducted in animal models. In rats, one study compared lymphatic recovery of DHA when provided in MAG (*n* = 4), DG (*n* = 5), TG (*n* = 6) and EE (*n* = 4) forms. Their results showed that MAG had about twice as much lymphatic recovery as TG or EE when the rats’ water supply was restricted [35]. Another study compared three types of DHA supplements in 80 rats, namely TG-DHA, MAG-DHA, and PL-DHA. Plasma recovery of DHA between the MAG and TG forms was similar over 60 days of supplementation but higher when provided as PL [36]. Erythrocyte recovery of DHA was greater with MAG and PL after 28 days of supplementation compared to TG [36]. Finally, all three types of supplementation increased % DHA in certain areas of the brain and retina after supplementation without any effect of the esterification form on the % DHA increment [36]. Generally, studies in rats support better lymphatic and blood recovery of MAG-DHA compared to the TG form. These findings from animal studies reinforce the idea that n-3 FA esterified in MAG increases blood n-3 FA levels to a greater extent without more being transferred to increase its level in organs and tissues.

The current study also found that females had a higher plasma concentration of EPA + DHA than males after the single dose intake of the EE form only. This may be caused by the female sex hormones that have been associated with an increase in plasma DHA [37], potentially by increasing the conversion of n-3 FA to long chain EPA and DHA [38]. This difference between sexes was not observed with the MAG form, potentially because of the higher levels of EPA and DHA and more inter-person variability that could have camouflaged the difference in endogenous synthesis between the two sexes.

Our study had both strengths and limitations. One of the strengths is the size of our sample, which allowed us to consider males and females separately. Few studies have compared the pharmacokinetics of n-3 FA between the two sexes. Also, we present the results in terms of relative % to other fatty acids as well as in absolute concentration. We found very few papers that present these two types of information. Relative % to other fatty acids makes it possible to obtain proportions of one fatty acid compared to other fatty acids while absolute concentrations provide a precise figure for the fatty acid quantity in the plasma. Thus, it is useful to have both units of measurement to get a better idea of the absolute vs. relative kinetics. One of the major limitations of this study is that EE was the comparison form provided instead of a TG form of n-3 FA. EE had previously been shown to be the least absorbed supplement.

## Conclusion

This study provides evidence that higher EPA and DHA plasma concentrations were reached when n-3 FA were provided esterified in MAG compared to the EE esterification form. Moreover, the MAG formulation had similar EPA and DHA plasma concentrations across males and females, which were reached when they were provided esterified in MAG rather than the EE supplement. This new evidence that the form of the FA influences n-3 FA plasma concentration provides an important framework for future studies, especially those addressing fat absorption problems.

## Supplementary information

supplemental material

## Data Availability

The datasets generated and/or analyzed during this study are available from the corresponding author on reasonable request.

## References

[1] Simopoulos AP (2002). The importance of the ratio of omega-6/omega-3 essential fatty acids. Biomed Pharmacother.

[2] Simopoulos AP (2016). An increase in the omega-6/omega-3 fatty acid ratio increases the risk for obesity. Nutrients..

[3] Stark KD, Aristizabal Henao JJ, Metherel AH, Pilote L (2016). Translating plasma and whole blood fatty acid compositional data into the sum of eicosapentaenoic and docosahexaenoic acid in erythrocytes. Prostaglandins, Leukotrienes Essent Fat Acids.

[4] Stark KD, Van Elswyk ME, Higgins MR, Weatherford CA, Salem N (2016). Global survey of the omega-3 fatty acids, docosahexaenoic acid and eicosapentaenoic acid in the blood stream of healthy adults. Prog Lipid Res.

[5] Hashimoto M, Hossain S (2011). Neuroprotective and ameliorative actions of polyunsaturated fatty acids against neuronal diseases: beneficial effect of docosahexaenoic acid on cognitive decline in Alzheimer’s disease. J Pharm Sci.

[6] Simopoulos AP (2002). Omega-3 fatty acids in inflammation and autoimmune diseases. J Am Coll Nutr.

[7] Svennerholm L (1968). Distribution and fatty acid composition of phosphoglycerides in normal human brain. J Lipid Res.

[8] Gomez-Pinilla F, Tyagi E (2013). Diet and cognition: interplay between cell metabolism and neuronal plasticity. Curr Opin Clin Nutr Metab Care.

[9] van der Lee SJ, Teunissen CE, Pool R, Shipley MJ, Teumer A, Chouraki V (2018). Circulating metabolites and general cognitive ability and dementia: evidence from 11 cohort studies. Alzheimer’s Dement.

[10] Hu FB, Bronner L, Willett WC, Stampfer MJ, Rexrode KM, Albert CM (2002). Fish and omega-3 fatty acid intake and risk of coronary heart disease in women. JAMA..

[11] Kris-Etherton Penny M, Harris William S, Appel Lawrence J (2002). Fish consumption, fish oil, omega-3 fatty acids, and cardiovascular disease. Circulation..

[12] Bhatt DL, Steg PG, Miller M, Brinton EA, Jacobson TA, Ketchum SB (2019). Cardiovascular risk reduction with icosapent ethyl for hypertriglyceridemia. N Engl J Med.

[13] von Schacky C, Harris WS (2007). Cardiovascular benefits of omega-3 fatty acids. Cardiovasc Res.

[14] Sanders TA, Hinds A, Pereira CC (1989). Influence of n-3 fatty acids on blood lipids in normal subjects. J Intern Med Suppl.

[15] Ghasemifard S, Turchini GM, Sinclair AJ (2014). Omega-3 long chain fatty acid “bioavailability”: a review of evidence and methodological considerations. Prog Lipid Res.

[16] Schuchardt JP, Hahn A (2013). Bioavailability of long-chain omega-3 fatty acids. Prostaglandins, Leukotrienes Essent Fat Acids.

[17] Chang CH, Tseng PT, Chen NY, Lin PC, Lin PY, Chang JP (2018). Safety and tolerability of prescription omega-3 fatty acids: a systematic review and meta-analysis of randomized controlled trials. Prostaglandins, Leukotrienes, Essent Fat Acids.

[18] Cruz-Hernandez C, Destaillats F, Thakkar SK, Goulet L, Wynn E, Grathwohl D (2016). Monoacylglycerol-enriched oil increases EPA/DHA delivery to circulatory system in humans with induced lipid malabsorption conditions. J Lipid Res.

[19] Folch J, Lees M, Sloane, Stanley GH (1957). A simple method for the isolation and purification of total lipides from animal tissues. J Biol Chem.

[20] Schuchardt JP, Schneider I, Meyer H, Neubronner J, von Schacky C, Hahn A (2011). Incorporation of EPA and DHA into plasma phospholipids in response to different omega-3 fatty acid formulations—a comparative bioavailability study of fish oil vs. krill oil. Lipids Health Dis.

[21] Lawson LD, Hughes BG (1988). Human absorption of fish oil fatty acids as triacylglycerols, free acids, or ethyl esters. Biochem Biophys Res Commun.

[22] Lawson LD, Hughes BG (1988). Absorption of eicosapentaenoic acid and docosahexaenoic acid from fish oil triacylglycerols or fish oil ethyl esters co-ingested with a high-fat meal. Biochem Biophys Res Commun.

[23] Davidson MH, Johnson J, Rooney MW, Kyle ML, Kling DF (2012). A novel omega-3 free fatty acid formulation has dramatically improved bioavailability during a low-fat diet compared with omega-3-acid ethyl esters: The ECLIPSE (Epanova® compared to Lovaza® in a pharmacokinetic single-dose evaluation) study. J Clin Lipidol.

[24] Galli C, Maggi FM, Risé P, Sirtori CR (2012). Bioequivalence of two omega-3 fatty acid ethyl ester formulations: a case of clinical pharmacology of dietary supplements. Br J Clin Pharmacol.

[25] Vandal M, Freemantle E, Tremblay-Mercier J, Plourde M, Fortier M, Bruneau J (2008). Plasma omega-3 fatty acid response to a fish oil supplement in the healthy elderly. Lipids..

[26] Plourde M, Vohl M-C, Vandal M, Couture P, Lemieux S, Cunnane SC (2009). Plasma n-3 fatty acid response to an n-3 fatty acid supplement is modulated by apoE ɛ4 but not by the common PPAR-α L162V polymorphism in men. Br J Nutr.

[27] Buttet M, Traynard V, Tran TTT, Besnard P, Poirier H, Niot I (2014). From fatty-acid sensing to chylomicron synthesis: role of intestinal lipid-binding proteins. Biochimie..

[28] D’Aquila T, Hung YH, Carreiro A, Buhman KK (2016). Recent discoveries on absorption of dietary fat: presence, synthesis, and metabolism of cytoplasmic lipid droplets within enterocytes. Biochim Biophys Acta.

[29] Plourde M, Chouinard-Watkins R, Vandal M, Zhang Y, Lawrence P, Brenna JT (2011). Plasma incorporation, apparent retroconversion and β-oxidation of 13C-docosahexaenoic acid in the elderly. Nutr Metab.

[30] Plourde M, Chouinard-Watkins R, Rioux-Perreault C, Fortier M, Dang MTM, Allard M-J (2014). Kinetics of 13C-DHA before and during fish-oil supplementation in healthy older individuals. Am J Clin Nutr.

[31] Léveillé P, Chouinard-Watkins R, Windust A, Lawrence P, Cunnane SC, Brenna JT (2017). Metabolism of uniformly labeled 13C-eicosapentaenoic acid and 13C-arachidonic acid in young and old men. Am J Clin Nutr.

[32] Djoussé L, Akinkuolie AO, Wu JHY, Ding EL, Gaziano JM (2012). Fish consumption, omega-3 fatty acids and risk of heart failure: a meta-analysis. Clin Nutr.

[33] James MJ, Cleland LG, James MJ (1997). Dietary n-3 fatty acids and therapy for rheumatoid arthritis. Semin Arthritis Rheumatism.

[34] Offman E, Marenco T, Ferber S, Johnson J, Kling D, Curcio D (2013). Steady-state bioavailability of prescription omega-3 on a low-fat diet is significantly improved with a free fatty acid formulation compared with an ethyl ester formulation: the ECLIPSE II study. Vasc Health Risk Manag.

[35] Banno F, Doisaki S, Shimizu N, Fujimoto K (2002). Lymphatic absorption of docosahexaenoic acid given as monoglyceride, diglyceride, triglyceride, and ethyl ester in rats. J Nutr Sci Vitaminol.

[36] Destaillats F, Oliveira M, Bastic Schmid V, Masserey-Elmelegy I, Giuffrida F, Thakkar SK (2018). Comparison of the incorporation of DHA in circulatory and neural tissue when provided as triacylglycerol (TAG), monoacylglycerol (MAG) or phospholipids (PL) provides new insight into fatty acid bioavailability. Nutrients..

[37] Giltay EJ, Gooren LJ, Toorians AW, Katan MB, Zock PL (2004). Docosahexaenoic acid concentrations are higher in women than in men because of estrogenic effects. Am J Clin Nutr.

[38] Childs CE, Hoile SP, Burdge GC, Calder PC (2012). Changes in rat n-3 and n-6 fatty acid composition during pregnancy are associated with progesterone concentrations and hepatic FADS2 expression. Prostaglandins, Leukotrienes Essent Fat Acids.

