# Supplementary material for: Comparison of pharmacokinetics of omega-3 fatty acid supplements in monoacylglycerol or ethyl ester in humans: a randomized controlled trial
Source: Eur J Clin Nutr. 2020 Oct 3;75(4):680–8. doi: 10.1038/s41430-020-00767-4 (PMC8035073; doi:10.1038/s41430-020-00767-4)
Supplement: Supplementary file 1 — supplemental material [file 41430_2020_767_MOESM1_ESM.pdf]

Supplemental file 1: Pharmacokinetic parameters of eicosapentaenoic acid and docosahexaenoic acid after monoacylglycerol and ethyl ester supplementation

PK parameters	Concentration EPA		Concentration DHA		% area EPA		% area DHA	
	Mean ± SD	P- value	Mean ± SD	P- value	Mean ± SD	P- value	Mean ± SD	P- value
Cmax								
- EE	1.99 ± 1.20	<0.0001	1.6 ± 0.9	<0.0001	0.65 ± 0.34	<0.0001	0.28 ± 0.16	<0.0001
- MAG	5.92 ± 2.47		4.1 ± 1.5		1.98 ± 0.76		1.30 ± 0.67	
Conc T=24h								
- EE	0.96 ± 0.73	<0.0001	0.4 ± 0.8	0.6614	0.36 ± 0.17	0.0034	0.05 ± 0.25	0.0136
- MAG	1.98 ± 0.71		0.5 ± 0.8		0.84 ± 0.23		0.20 ± 0.18	
AUC 0-5h								
- EE	1.4 ± 1.1	<0.0001	1.9 ± 1.5	<0.0001	0.50 ± 0.28	<0.0001	0.40 ± 0.41	0.001
- MAG	7.3 ± 2.5		5.7 ± 2.0		2.51 ± 0.87		1.91 ± 0.93	
AUC 0-24h								
- EE	24.5 ± 6.9	<0.0001	13.8 ± 7.11	<0.0001	8.01 ± 1.62	<0.0001	1.43 ± 1.26	<0.0001
- MAG	62.4 ± 9.7		27.2 ± 8.8		22.89 ± 2.84		8.32 ± 2.14	

PK = Pharmacokinetic, EPA= Eicosapentaenoic acid, DHA = Docosahexaenoic acid, EE = Ethyl ester, MAG = Monoacylglycerol, SD = standard deviation, Cmax = maximum concentration, Conc T=24h = Concentration of FAs at 24h post supplementation, AUC 0-5h = Area under the curve from time 0 to 5h post supplementation, AUC 0-24h = Area under the curve from time 0 to 24h post supplementation

Supplemental file 2: Pharmacokinetic parameters of eicosapentaenoic acid + docosahexaenoic acid in males and females after monoacylglycerol and ethyl ester supplementation.

Pharmacokinetic parameters	EE		MAG	
	Mean ± SD	P- value	Mean ± SD	P- value
Cmax				
- Males	2.55 ± 1.97	0.0295	9.12 ± 4.83	0.5268
- Females	4.45 ± 1.60		10.31 ± 3.20	
Concentration T=24h				
- Males	0.96 ± 1.07	0.2080	2.52 ± 1.43	0.0567
- Females	1.70 ± 1.46		2.38 ± 0.97	
AUC 0-5h				
- Males	1.8 ± 1.8	0.0111	11.6 ± 4.9	0.1547
- Females	4.8 ± 2.8		14.4 ± 3.4	
AUC 0-24h				
- Males	27.4 ± 11.9	0.0006	90.1 ± 20.7	0.8941
- Females	49.6 ± 11.8		89.1 ± 13.2	

EE = Ethyl ester, MAG = Monoacylglycerol, SD = standard deviation, Cmax = maximum concentration, Concentration T=24h = Concentration of FAs at 24h post supplementation, AUC 0-5h = Area under the curve from time 0 to 5h post supplementation, AUC 0-24h = Area under the curve from time 0 to 24h post supplementation
